# Development-dependent changes in the tight DNA-protein complexes of barley on chromosome and gene level

**DOI:** 10.1186/1471-2229-9-56

**Published:** 2009-05-12

**Authors:** Tatjana Sjakste, Kristina Bielskiene, Marion Röder, Olga Sugoka, Danute Labeikyte, Lida Bagdoniene, Benediktas Juodka, Yegor Vassetzky, Nikolajs Sjakste

**Affiliations:** 1 Genomics and Bioinformatics, Institute of Biology, University of Latvia, Miera 3, LV2169 Salaspils, Latvia; 2 Department of Biochemistry and Biophysics, Vilnius University, M. K. Čiurlionio 21, LT2009 Vilnius, Lithuania; 3 Gene and Genome Mapping, Leibniz Institute of Plant Genetics and Crop Plant Research, Correnstrasse 3, 06466, Gatersleben, Germany; 4 UMR-8126, Institut Gustave Roussy, 39, rue Camille-Desmoulins, 94805 Villejuif, France; 5 Faculty of Medicine, University of Latvia, Šarlotes 1a, LV1001, Riga, Latvia

## Abstract

**Background:**

The tightly bound to DNA proteins (TBPs) is a protein group that remains attached to DNA with covalent or non-covalent bonds after its deproteinisation. The functional role of this group is as yet not completely understood. The main goal of this study was to evaluate tissue specific changes in the TBP distribution in barley genes and chromosomes in different phases of shoot and seed development. We have: 1. investigated the TBP distribution along *Amy32b *and *Bmy1 *genes encoding low pI α-amylase A and endosperm specific β-amylase correspondingly using oligonucleotide DNA arrays; 2. characterized the polypeptide spectrum of TBP and proteins with affinity to TBP-associated DNA; 3. localized the distribution of DNA complexes with TBP (TBP-DNA) on barley 1H and 7H chromosomes using mapped markers; 4. compared the chromosomal distribution of TBP-DNA complexes to the distribution of the nuclear matrix attachment sites.

**Results:**

In the *Amy32b *gene transition from watery ripe to the milky ripeness stage of seed development was followed by the decrease of TBP binding along the whole gene, especially in the promoter region and intron II. Expression of the *Bmy1 *gene coupled to ripening was followed by release of the exon III and intron III sequences from complexes with TBPs. Marker analysis revealed changes in the association of chromosome 1H and 7H sites with TBPs between first leaf and coleoptile and at Zadoks 07 and Zadoks 10 stages of barley shoot development. Tight DNA-protein complexes of the nuclear matrix and those detected by NPC-chromatography were revealed as also involved in tissue- and development-dependent transitions, however, in sites different from TBP-DNA interactions. The spectrum of TBPs appeared to be organ and developmental-stage specific. Development of the first leaf and root system (from Zadoks 07 to Zadoks 10 stage) was shown as followed by a drastic increase in the TBP number in contrast to coleoptile, where the TBPs spectrum became poor during senescence. It was demonstrated that a nuclear protein of low molecular weight similar to the described TBPs possessed a high affinity to the DNA involved in TBP-DNA complexes.

**Conclusion:**

Plant development is followed by redistribution of TBP along individual genes and chromosomes.

## Background

Reorganization of the chromatin structure is one of the main mechanisms for regulation of gene expression in plants, chromatin rearrangements take place in response to light and tissue-specific signalling molecules [1]. Despite rapid progress in the field, the functional significance of some groups of nuclear proteins, including the tightly bound to DNA proteins (TBP), remains obscure. TBP is a specific nuclear protein group that remains attached to DNA with covalent or non-covalent bonds after its deproteinisation independently of the deproteinisation method applied: protease digestion, phenol extraction, chloroform extraction or salting-out. The TBP have been found in the DNA of numerous evolutionary distant species [2]. Both the function of the TBPs and the nature of DNA sequences involved in the tight complexes remain to be detailed. Enrichment of the TBPs in specific DNA sequences is of special interest in connection with speculation on the potential function of such sequences in higher order structures of the genome of different organisms including humans, mouse, and chicken [3-6]. In our recent work [7] we have applied the DNA microarray technique to study the distribution of TBPs along the chicken alpha-globin domain in cell lines that expressed the gene, did not express it or conducted abortive expression. In this study we have shown profound transcription-dependent changes in the TBP-distribution pattern in the alpha-globin domain. Other preliminary results from of our team [8] also indicated the existence of tissue- and development specificity in the patterns of TBP distribution in barley (*Hordeum vulgare*) shoots.

The life cycle of the barley plant, especially the shoot and seed provide an excellent model for plant development studies. Barley shoot and seed developmental stages are well characterized and classified [9]. Etiolated barley seedlings provide cell populations with different proliferation, differentiation and senescence status including synchronously dividing cell populations of primary leaf [10-12], senescent coleoptiles and a mixed cell population from roots [10]. In the present work we used dry grains (Zadoks 0) and grains after 20 h of imbibition (Zadoks 1); coleoptiles, first leaves and roots were dissected from shoots of Zadoks 07 (coleoptile emerged stage, classification according to [9]) and Zadoks 10 (first leaf through coleoptile) development stages as well as seeds of watery ripe (Zadoks 71) and medium milk development (Zadoks 75).

Moreover, expression of several specific enzymes of carbohydrate metabolism in barley is development- or/and tissue specific and is restricted to well-defined stages of the plant development [13]. It was reported that transcription of α-amylase genes is low and decreases during seed development, but β-amylase expression in endosperm is up-regulated during its development [13-15]. Two representatives of each group of amylase genes, the *Amy32b *and *Bmy1 *genes provide prospective model systems. Highly conserved in barley cultivars, *Amy32b *gene [16] is located on chromosome 7H in the centromere region [17]. Gene encodes a low-pI α-amylase and is expressed in barley aleurone cells under the control of gibberellic acid and abscisic acid [15]. Highly polymorphic in structure, barley *Bmy1 *gene [18,19] is located in the long arm of chromosome 4H [20], encodes endosperm specific β-amylase and is expressed only during seed development [18]. Several allelic forms of *Bmy1 *structural gene were sequenced and analyzed in relation to their functionality [21-24].

Despite the fact that barley genome is not completely sequenced, work with this plant provides the opportunity to use the technique of mapped barley genomic markers developed during recent decades [25,26]. The tool allows chromosome profiling to be performed in any application.

In order to study transcription, tissue and development dependent changes in TBPs distribution in barley genes and chromosomes at different phases of shoot and seed development, we have formulated the following as the goals of the present study: 1. to investigate the distribution of TBPs along *Amy32b *and *Bmy1 *genes using oligonucleotide DNA arrays; 2. to characterize the polypeptide spectrum of TBP in different shoot organs and during different development stages. 3. to apply mapped barley MS as a tool to study tissue and developmental specificity in the distribution of DNA complexes with TBP (TBP-DNA) in the barley chromosomes 1H and 7H; 4. to compare the chromosomal distribution of TBP-DNA complexes and distribution of tight DNA-protein complexes separated using other than TBP isolation approaches including nuclear matrix isolation and nucleoprotein chromatography on celite (NPC-chromatography).

## Results

### RT-PCR

**Figure 1:**
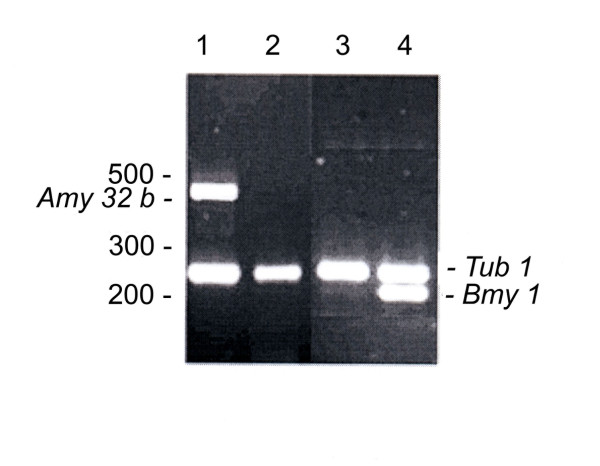
***Amy32b *(lanes 1, 2), *Bmy1 *(lane 3, 4) and alpha tubulin (lanes 1 – 4) RT-PCR products**. Lanes 1, 3 – RNA from watery ripe seeds; lanes 2, 4 – RNA from milky ripe barley seeds. Positions of the molecular weight markers (bp) are indicated on the right. Positions of RT-PCR products are indicated on both sides of the figure.

Figure 1 presents data on *Amy32b *(lanes 1, 2) and *Bmy1 *(lanes 3, 4) genes expression in seeds of watery ripe (Zadoks 71, lanes 1, 3) and medium milk (Zadoks 75, lanes 2, 4) stages of development. Reference gene *Tub1*, a ubiquitous and stably expressed gene in barley, was expressed with the same intensity at both stages analyzed. It was found that *Amy32b *gene is highly expressed in watery ripe and silent in milky ripe seeds. On the contrary, expression of the *Bmy1 *gene was detected exclusively in the milky ripe stage. Thus, the chosen barley genes at two stages of seed development represented a model system to be used in further analysis of TBPs distribution along the silent and expressed genes.

### Microarray analysis

**Figure 2:**
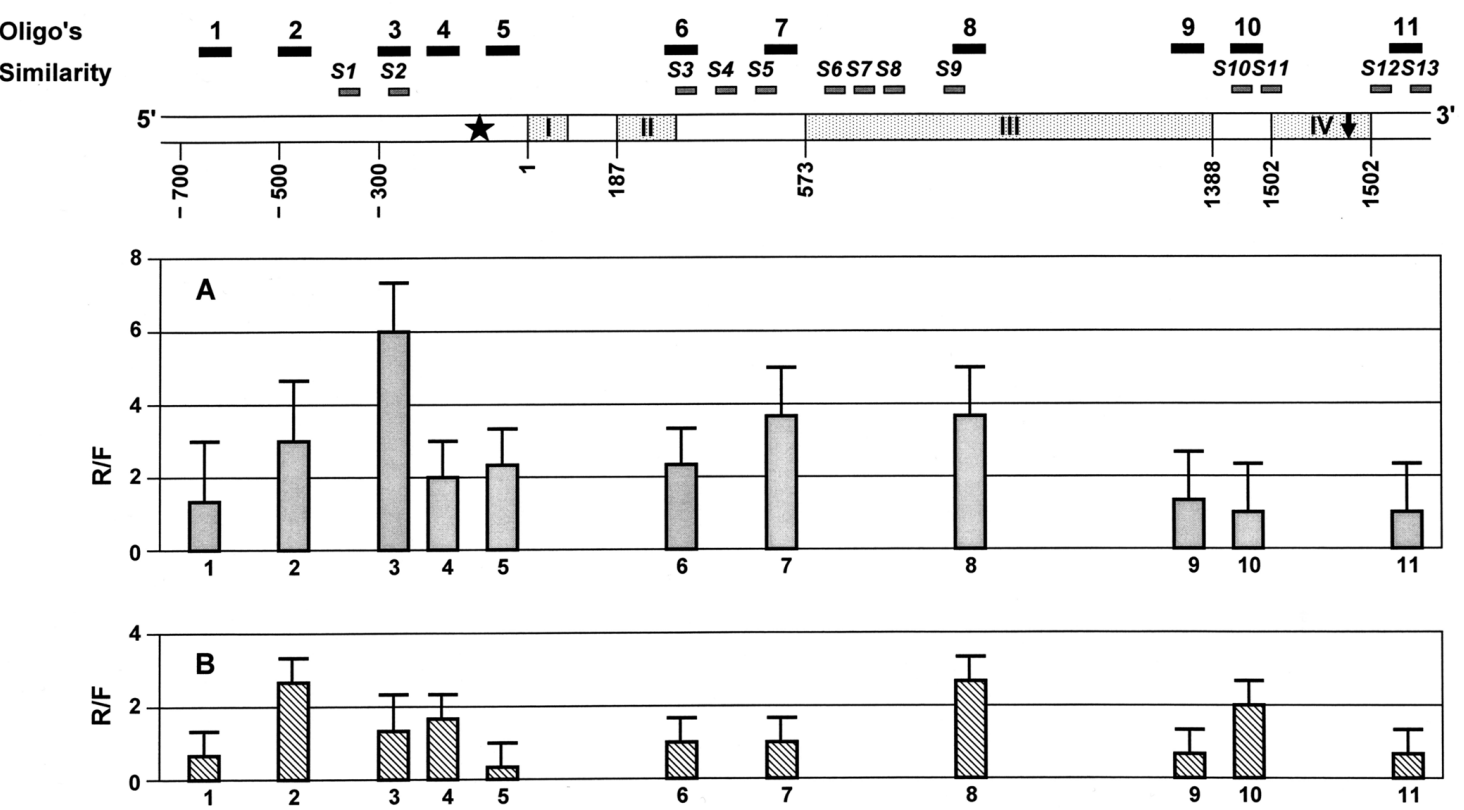
**DNA array based mapping of the TBPs distribution in *Amy32b *structural gene in watery ripe (A) and milky ripe (B) barley seeds**. Upper panel presents the gene structure with positions of oligonucleotides of the array and regions of the similarity with *Bmy1 *(S1 – S13). Star indicates position of the TATA box, black arrow in last exon indicates stop codon. Exon numbers are given as Roman numerals. The data in lower panels represent the ratio of hybridization of R vs F DNA fractions scored as an average of three independent experiments (two hybridizations per experiment). Error bars represent standard deviation.

**Figure 3:**
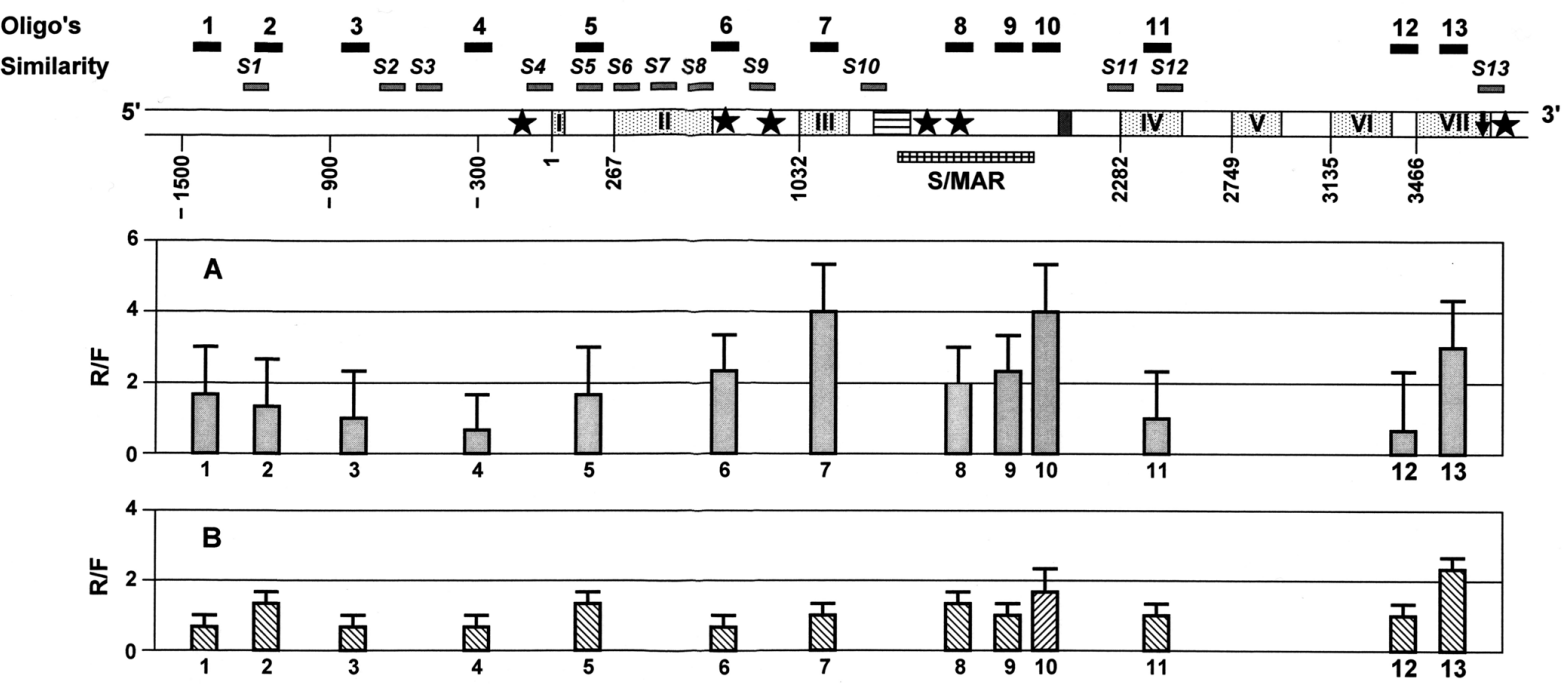
**DNA array based mapping of the TBPs distribution in *Bmy1 *structural gene in watery ripe (A) and milky ripe (B) barley seeds**. Upper panel presents the gene structure with positions of oligonucleotides of the array and regions of similarity with *Amy32b *gene (S1 – S13). Stars correspond to the positions of the TATA boxes; black arrow in last exon VII indicates the stop codon. Exon numbers are given in Roman numerals. Black and dashed squares in the Intron III indicate the positions of the microsatellite and MITE element, correspondingly. The predicted MAR position is indicated by a checked bar. The data on lower panels represent the ratio of hybridization of R vs F DNA fractions scored as an average of three independent experiments (two hybridizations per experiment). Error bars represent standard deviation.

Figure 2 and Figure 3 present quantified results of hybridization between TBP-DNA complexes fractionated from seeds of watery ripe (Zadoks 71, panels A) and medium milk (Zadoks 75, panels B) stages and DNA microarray of *Amy32b *(Figure 2) and *Bmy 1 *(Figure 3) genes correspondingly. In samples from watery ripe seeds, ratios of hybridization intensities with R probe (probe derived from the DNA complexed with tightly bound proteins) and F probe (probe derived from the TBP-free DNA) and *Amy32b *microarray were approximately the same in oligonucleotide positions 1, 9, 10 and 11 reflecting an equal or similar amount of the corresponding DNA fragments in both TBP bound and TBP unbound fractions. In all other positions the ratio exceeded 1, indicating the enrichment in TBPs in the corresponding DNA fragments. Positions 3 (300 bp upstream the translation start codon), 7 (downstream part of Intron 2), and 8 (Exon 3) were shown as the most TBP-enriched in samples from watery-ripe seeds. Transition to milky ripening was followed by the decrease of R vs F ratio in general along the whole *Amy32b *gene. Ratio much below 1 in positions 1, 5, 9 and 11 indicates predominant accumulation of corresponding DNA fragments in the TBP- free F fraction. In positions 3 and 7 the ratio decreased from 6 and 4 to almost 1, and from 4 to 2.5 in position 8. The position of oligonuclotide 10, in which the ratio increased from 1 to 2, was an exception. Thus, during seed development, an overall decrease in TBP-DNA interactions (R → F transition) occurs along the *Amy32b *gene being most drastic in the promoter and Intron 2 gene regions.

The R and F fractions obtained from watery ripe seeds hybridized also with different intensity with *Bmy 1 *gene microarray. An R vs F ratio close to or less than 1 was observed for positions 3 and 11, and 4 and 12 correspondingly. In positions 1, 2, 5, and 8 hybridization with R probes was somewhat stronger than with F probes, however the R vs F ratio did not exceed 2. The R vs F ratio was rather high in oligonucleotide positions 6, 9 (more than 2) and 13 (near to 3). Finally, in positions 7 and 10, the intensity of hybridization with R probe was 4 times stronger than with F probe. The highest ratio in the milky ripeness stage (Figure 3, panel B) was close to 2 in position 10, and slightly exceeded 2 in position 13. In all other oligonucleotide positions the R vs F ratio was around 1. Thus, similar to the results obtained with *Amy32b *gene, the overall decrease of the R vs F ratio is revealed along the *Bmy1 *gene during seed development. The process is more pronounced in Exon 3 and upstream the microsatellite locus in Intron III.

### Bioinformatic analysis of Amy32b and Bmy 1 gene sequences

Identification of specific areas of TBP binding in both genes raised the question of peculiarities in the gene sequences in these areas. A search for nuclear matrix attachment regions (MARs) performed by the MatInspector program (Rel. 7.4), revealed a possible MAR in Intron 3 of the *Bmy1 *gene (Figure 3). No MARs were detected in the *Amy32b *gene. DiAlign TF, Release 3.1 revealed 13 homology regions between the two genes (Figures 2 and 3), however no common features in the distribution of transcription factor binding sites along the genes and transcription factor binding modules were revealed. Comparison of the predicted MAR with the TBP binding sites in the *Bmy1 *gene Intron 3 indicates that this site is enriched in TBPs. Similar sequences of the two genes differed in their affinity to TBPs. The only exception was observed in the area of similarity S9; these sites were TBP-enriched in both genes.

### Profiling of DNA-protein complexes along chromosomes

Positive data on intragenic changes in TBP distribution in the course of development encouraged us to upscale the investigation and to study the long-range distribution of tightly bound proteins along barley chromosomes 1H and 7H. Lack of barley genome sequence information makes it impossible to apply microarray technology to study long-range distribution of TBPs along chromosomes. To at least partly reach the goal we have used the tool of mapped markers well developed in barley studies [25-27]. Profiling experiments were designed as PCR-based identification of the DNA matrices in DNA-protein complexes obtained by different fractionation approaches. Involvement of the DNA stretch in interactions with a given protein group (TBP, nuclear matrix) was scored in terms of the presence/absence of amplification with mapped markers of barley chromosomes 1H and 7H. Data are organized as graphical representations of chromosome 1H and 7H with an indication of the PCR result at each MS locus in all DNA-protein fractions analyzed. The study was performed on DNA from barley shoot organs; this model enables us to obtain a sufficient quantity of material for fractionation of nuclear structures.

### TBPs

**Figure 4:**
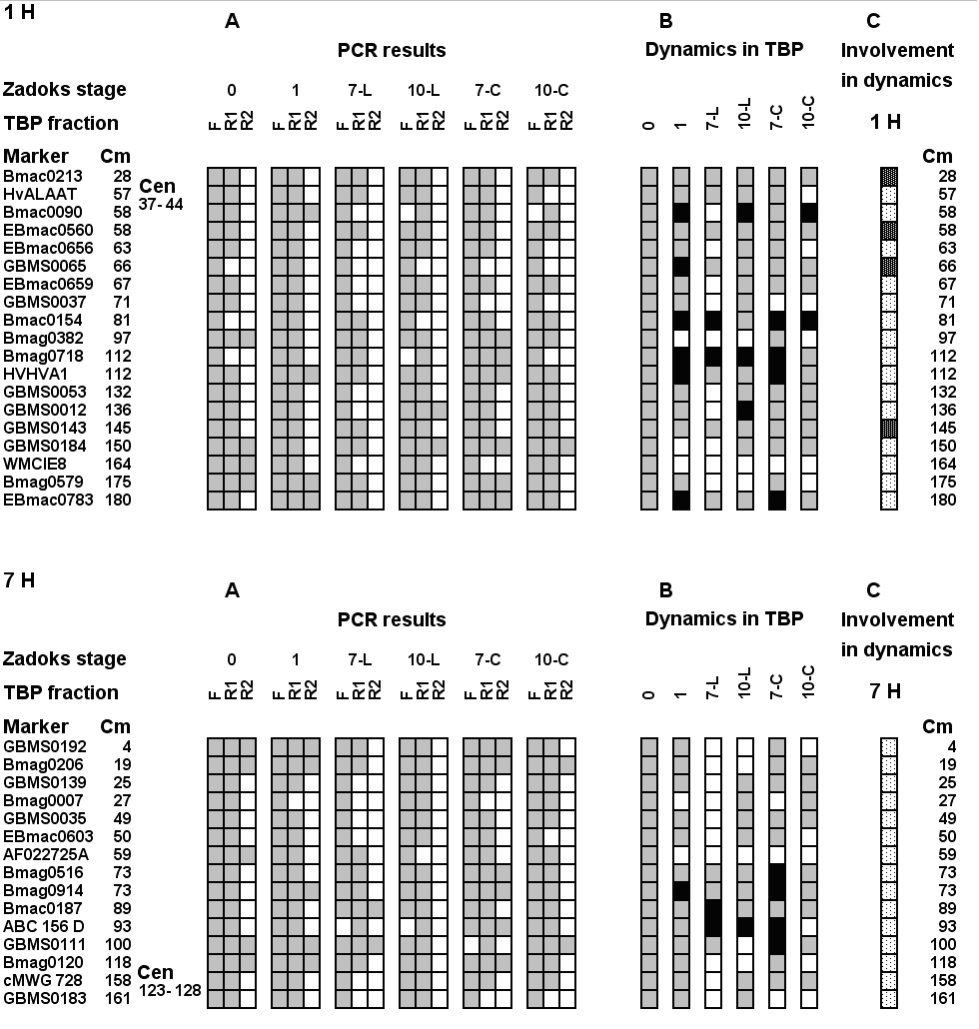
**The distribution of microsatellite sequences in free DNA and tight DNA-protein complexes along barley chromosomes 1H and 7H**. A. The distribution of DNA fragments containing given microsatellites in free DNA (F), and tight DNA-protein complexes (R1 and R2). Marker names are given in the left column, following column gives distance from the chromosome extremity in centimorganides. Position of the centromere is indicated separately (Cen). 0 – dry seeds (phase 0), 1 – 20 hours of imbibition (phase 01); 7-L – first leaf on stage 07; 10-L – leaf on stage 10; 7-C – coleoptile on stage 07; 10-C – coleoptile on stage 10. Grey squares – presence of amplification; white squares – absence of amplification. B. Summary of the trends of transitions during development of different organs compared with the situation in dry seed. Grey squares – situation in the seed or similar; white squares – decrease of association with TBPs; black squares – increase in TBP-DNA interactions. C. General summary of involvement of the studied genomic sites in R-F and adverse transitions. Black points on white background – site involved in transitions; white points on black background – site not involved in transitions.

Figure 4 presents graphically both chromosomes as profiles of TBP-DNA complexes in seeds before (Zadoks 0) and after imbibition (Zadoks 1), as well as leaves and coleoptiles at two stages of development (Zadoks 07 and 10). In dry seeds most markers of both chromosomes were found in F and R1 fractions. Only four and five markers of 1H and 7H correspondingly were present in the R2 fraction. Three markers of 1H (GBMS0065, Bmac0154, Bmag0718) were found exclusively in the filtered TBP-free DNA (Figure 4A). However, positive amplification in R1 was detected in the above loci after imbibitions. Positive amplification in the R2 fraction with Bmac0090, HVHVA1, and EBmac0783 of 1H and with Bmag0914 of 7H was also obtained only after imbibition. These results indicate an increase in the number of the tight interaction sites between TBPs and DNA. In contrast, the loci Bmag0382, GBMS0184 and WMCIE8 of 1H and AF022725A, cMWG 728 and GBMS0183 of 7H disappeared from the R2 fraction during transition from the Zadoks 0 to the Zadoks 1 stage. In stage 07 leaves some regions of both chromosomes appear to be free of TBPs. No markers of chromosome 1H and only two 7H markers were found in R2 in Zadoks 07 leaves. In Zadoks 07 leaves in largest number of loci interacting with TBPs was decreased when compared to dry seeds. Only two loci on Chromosome 1H (Bmac0154 and Bmag0718), formerly unbound to TBP became involved in complexes with TBP. Markers Bmac0187 and ABC 156 D of 7H appeared in R2 fraction and disappeared from F fraction correspondingly. The transition of the leaf to the Zadoks10 stage was followed by a substantial increase in stretches involved in complexes with TBPs when compared to Zadoks 07 stage (positive amplification appeared in R1 fractions at most loci). However, DNA templates were not detected in R2 fractions along both chromosomes except for the GBMS0012 and GBMS0184 loci of 1H. Interestingly, in stage 07 coleoptiles the number of sites involved in TBP-DNA interactions was somewhat greater than in dry seeds (most markers were found in the R1 fraction, several also in the R2 fraction). The transition to stage 10 in coleoptiles was followed a decrease in the number of genomic sites involved in interaction with TBPs. In the stage 10 coleoptiles only one marker of 1H and two markers of 7H were found in the R2 fraction, some markers disappeared from the R1 fraction.

Panel B in Figure 4 summarizes the trends of transitions during development of different organs compared with the situation in dry seed. Panel C gives a summary of the involvement of the Chromosome 1 H and 7H sites of the R ↔ F transitions. It emerges that all the sites on chromosome 7H are involved, and only four sites of 1H are not involved in the dynamics. Thus, the development of the barley seedling is coupled to rearrangements in the interactions of DNA with TBPs both on chromosome 1H and 7H. The process is organ specific and can be termed "tightening" of the DNA-TBP interactions in the leaves and "loosening" in the coleoptile compared to dry seed status.

### Chromatin fractions and nuclear matrix

**Figure 5:**
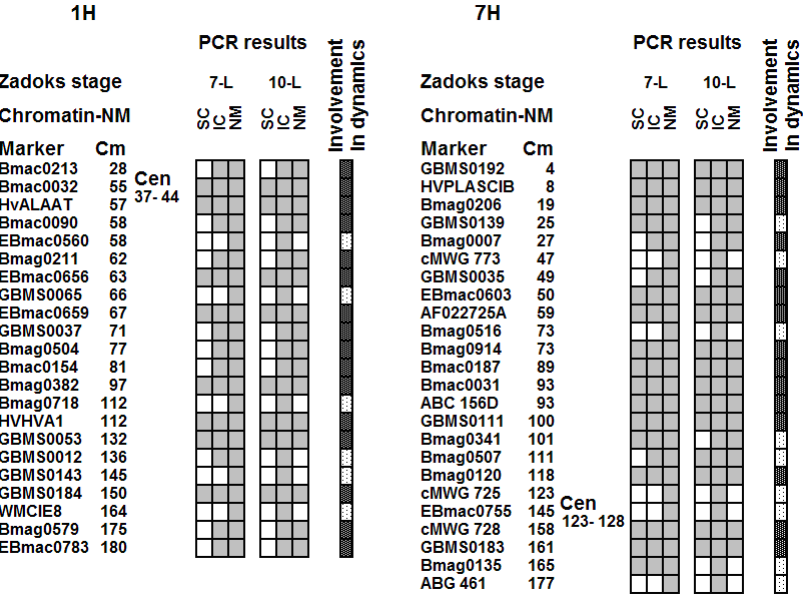
**The distribution of microsatellite sequences in chromatin fractions and nuclear matrix-attached DNA along barley chromosomes 1H and 7H**. Columns in the right part of the figure illustrate the distribution of DNA fragments containing given microsatellites in fractions of soluble chromatin (SC), insoluble chromatin (IC) and the nuclear matrix (NM). Panel on the right indicates involvement in association or dissociation of the nuclear matrix. Grey and white squares correspond to the presence and absence of amplification correspondingly. Black points on white background and white points on black background indicate on the sites involved and not involved in transitions. All other designations are as in Figure 4.

Figure 5 presents the distribution of molecular markers in DNS fragments in soluble chromatin (SC), insoluble chromatin (IC) and nuclear matrix (NM) fractions extracted from leaves at the Zadoks 07 and Zadoks 10 stages. In stage 07 leaves, the markers EBmac0560, GBMS0065, Bmag0718, GBMS0143, GBMS0012, WMCIE8 of 1H and markers cMWG773, Bmag0516, cMWG725, EBmac0755, ABG461 of 7H were found in the nuclear matrix-attached DNA. All these markers moved to the insoluble chromatin fraction at stage 10. Thus senescence of the first leaf appears to be accompanied by a "loosening" of the DNA-NM interactions accompanied by complete detachment of some chromosomal regions of the nuclear matrix. The right panel of the Figure 5 indicates the sites involved in transitions in DNA-chromatin and DNA-NM interactions. Interestingly, data obtained on both chromosomes indicate that in our model many fewer chromosomal sites appear to be involved in the transitions of DNA -NM interactions compared to the number of R ↔ F transition loci revealed by the profiling of the TBP-DNA complexes (Figure 4A, D; Figure 5).

### NPC chromatography

**Figure 6:**
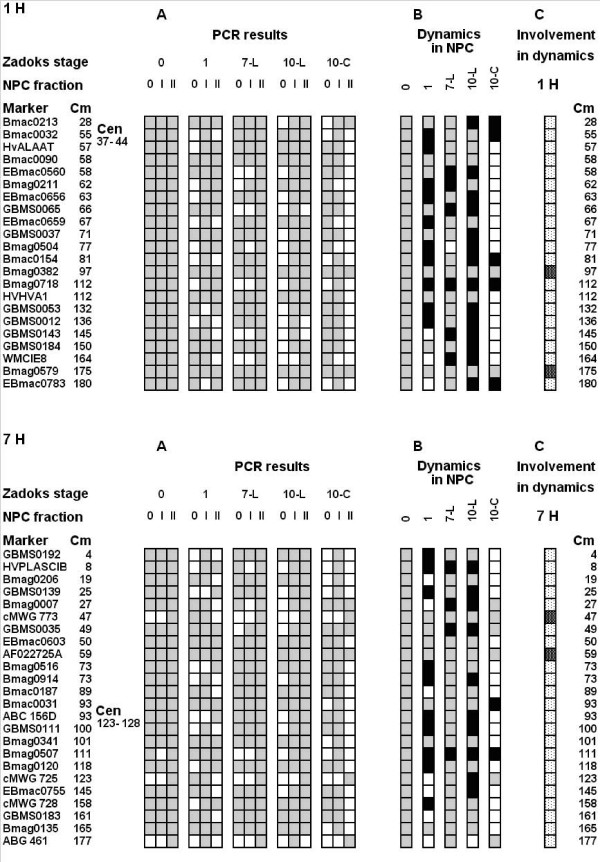
**The distribution of microsatellite sequences in fractions obtained in the course of chromatography of nucleoproteins on celite**. A. The distribution of DNA fragments containing given microsatellites in unbound to nuclear matrix fraction (eluted in NaCl gradient, DNA 0, column 0 on the Figure), loosely bound to the nuclear matrix (eluted in LiCl-urea gradient and in temperature gradient below 70°C, DNA I (column I) and tightly bound to the nuclear matrix (eluted in temperature gradient at 90°C, DNA II, column II). Grey and white squares indicate presence and absence of amplification correspondingly. All other designations are like in Figure 4. B. Summary of the trends of transitions during development of different organs compared with the situation in dry seed. Grey squares – situation in the seed or similar; white squares – loosening of association with the nuclear matrix; black squares -tightening of interactions. C. General summary of involvement of the studied genomic sites in association-dissociation according the NPC chromatography data. Black points on white background – sites involved in transitions; white points on black background – sites not involved in transitions.

The nucleoprotein chromatography method enables the discrimination of several types of DNA-protein interactions in the nucleus including two kinds of DNA complexes with nuclear matrix proteins that are not revealed by other approaches. In this method cell lysate is loaded onto a celite column. Celite irreversibly binds all proteins including protein moieties of the nucleoproteins. Nucleic acids are gradually released from the protein complexes by sequential gradients of NaCl, LiCl-urea and temperature. DNA not bound to the nuclear matrix is eluted in a NaCl gradient (DNA 0), DNA "loosely" bound to the nuclear matrix is released using a LiCl-urea gradient (DNA I), high temperature is necessary to release DNA from the strong complexes with the nuclear matrix (DNA II). We suppose that single strand DNA breaks in the vicinity of the replication complex induce the transition DNAII – DNA I, but double strand breaks release the DNA0 fraction [28,29]. Graphical presentations of chromosomes 1H and 7H (Figure 6) illustrate the results of amplification on the DNA purified from the different NPC-fractions: DNA 0, DNA I and DNA II [28,29]. In dry seeds all the 1H markers and most of the 7H markers were revealed in all three chromatographically analyzed fractions. Data obtained on both chromosomes, reflect complex patterns of chromatin domain reorganizations after imbibitions. Among 22 loci tested on 1H, six disappeared from the DNA II fraction (Figure 6, 1H A and B). Among 24 markers used in 7H profiling, 11 became involved in looser interactions with the nuclear matrix (Figure 6, 7H, A and B). Development of the first leaf (Zadoks 07) was accompanied by an increase in loci number associated with the nuclear matrix including EBmac0560, Bmag0211, GBMS0065, Bmag0718, GBMS0143, WMCIE8 and HVPLASCIB, Bmag0007, GBMS0035, Bmag0507 for 1H and 7H correspondingly. Several loci became unbound to NM via the strong bond (GBMS0065, GBMS0012, GBMS0143 and ABG 461 for 1H and 7H correspondingly) at the Zadoks 10 stage. However, in general, senescence of the first leaf was followed by an increase in the sites interacting with the nuclear matrix (Figure 6, Panel B). Interestingly, in Zadoks10 coleoptiles the profiles of both chromosomes indicate detachment of DNA from nuclear matrix or loosening of the DNA-NM bonds in most loci tested. Only three (Bmac0213, Bmag0382, Bmag0579) and six (Bmag007, cMWG 773, GBMS0035, AF022725A, cMWG 725, ABG 461) loci from 22 and 24 tested correspondingly for 1H and 7H chromosomes remained tightly attached to the nuclear matrix. Most sites of both chromosomes were involved in the rearrangements with two exceptions for each chromosome. (Figure 6, Panel C).

### Tightly bound protein spectrum in organs of stage 07 and stage 10 shoots

**Figure 7:**
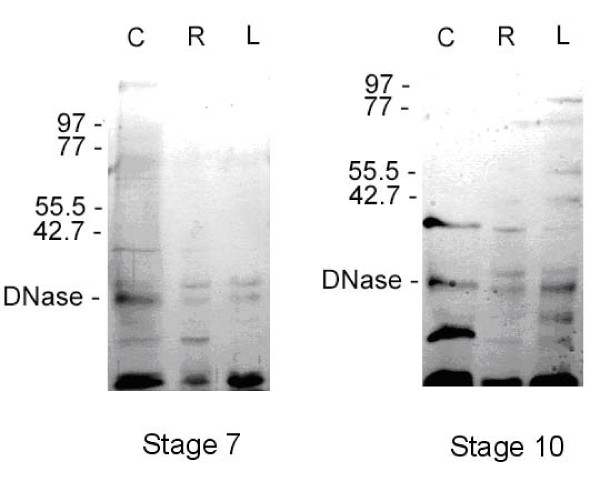
**Electropherograms of the tightly bound proteins obtained by DNase I digestion of bulk DNA of leaves (L), roots (R) and coleoptiles (C) of stage 07 and stage 10 barley shoots**. Arrows indicate positions of molecular weight markers (KDa). 10% PAAG. Silver staining.

Figure 7 presents electropherograms of the TBPs obtained from the leaves, roots and coleoptiles of Zadoks stage 07 and Zadoks stage 10 shoots using method of exhaustive DNase digestion. Amazingly, the TBP spectrum appears to be organ and developmental stage-specific. In stage 07 leaves there is only one TBP of molecular weight 30 KDa, and the transition to stage 10 and senescence of the leaf is followed by a drastic increase of the TBP number, additional bands of 17, 21, 36, 42, 55, 60, 69 and 76 KDa are clearly visible. And a similar process happens in the roots. Besides the 30 KDa band a smaller 20 KDa polypeptide is detected in young roots. Additional 36 KDa protein and some minor high-molecular polypeptides are detectable in old roots. Changes in the TBP spectrum in coleoptile appear to be inversed. The TBP pattern in stage 07 coleoptile is rather complicated. There is a high molecular weight polypeptide of about 200 KDa, bands at 67, and 38 KDa, as well as a 30 KDa band common for all organs and a 20 KDa band found also in the roots. In contrast, only two polypeptides are found in stage 10 coleoptile, the 36 KDa polypeptide, detected in all organs of stage 10 shoots and 20 KDa, also found in all organs, but it seems to be prominent in the coleoptile. Thus the polypeptide spectrum of TBPs appears to depend on the plant organ development stage. Further sets of experiments were performed to reveal specificity of interactions of TBPs with DNA sequences.

### Affinity of the TBP-bound DNA to nuclear proteins

**Figure 8:**
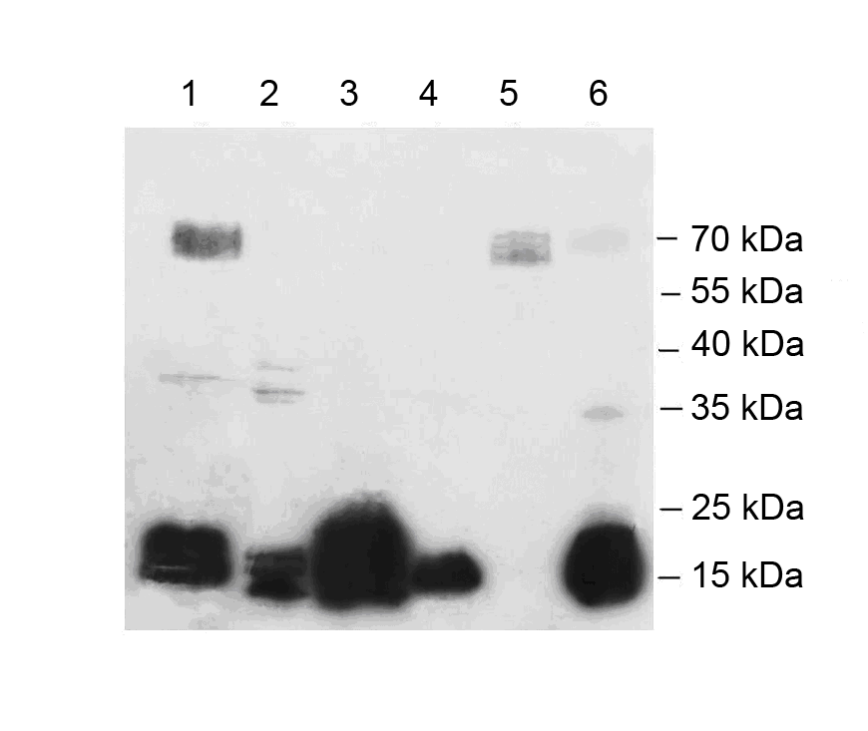
**DNA-binding protein blot assay**. Assay was performed with electrophoretically fractionated nuclear proteins isolated from the Zadoks 07 (1 – 3) and Zadoks 10 (4 – 6) coleoptiles (1, 4), leaves (2, 5), and roots (3, 6) and incubated with TBP-associated DNA from Zadoks 10 leaves.

To test changes in the tight DNA-protein interactions coupled to plant development we performed DNA-binding protein blot assays with electrophoretically fractionated nuclear polypeptides extracted from different barley organs at development stages 07 and 10 and TBP-bound DNA from the stage 10 leaves. Results are shown in Figure 8. Nuclear polypeptides isolated from the stage 07 coleoptiles manifested high affinity to the TBP-associated DNA. Both low-molecular weight and 70 kDa peptides formed tight complexes with DNA. Transition to stage 10 was associated with a decrease of DNA-binding proteins, only low-molecular weight proteins could bind to the probe (Figure 8, lanes 1 and 4). Mostly low molecular weight polypetides extracted from stage 07 leaves manifested affinity to the probe, in stage 10 leaves these were replaced by 70 kDa peptides (Figure 8, lanes 2 and 5). The TBP-associated DNA probe recognized low molecular nuclear polypeptides extracted from the stage 07 roots, in extracts from stage 10 roots binding to these polypeptides became less intense, however additional 35 kDa and 70 kDa polypeptides with affinity to the probe were detected.

## Discussion

### Gene expression changes during seed development

In the present study we have revealed development-dependent changes of the TBP distribution in *Amy32b *and *Bmy1 *genes during transition of the barley seed from watery-ripe to middle milk ripe stage. Changes in TBP distribution in the genes were coupled to changes in their expression. Our RT-PCR data confirm earlier published data indicating that β-amylase expression is linked to starchy endosperm development, but α-amylase is not expressed on late seed development stages [14], moreover over-expression of α-amylase in developing seed leads to development defects [15].

### TBP redistribution in the Amy32b gene

Our observation of a decrease in TBP interactions with promoter region of the *Amy32b *gene is in good agreement with a recently proposed hypothesis [13]. According to this hypothesis in the dormant gene negative regulators bind to the corresponding cis-acting elements in the promoter and form a "repressosome," this diminishes the binding or transactivating activities of positive regulators to the promoter, thereby preventing *Amy32b *transcription. Induction of transcription is followed by binding of positive regulators to their respective DNA sequences and formation of the "enhanceosome," leading to a high level of *Amy32b *gene expression. Probably, formation of the "enhanceosome" is preceded by degradation of repressors. Taking into account that the RT-PCR data expression of the gene is observed exclusively in the watery ripeness stage, one can speculate that TBPs could stabilize "enhanceosome", release or degradation of TBPs could be one of the mechanisms of the promoter inactivation. Interestingly in the dormant *Bmy 1*gene the 300 bp region upstream the translation start codon (position 4) is neither TBP-enriched. The very organization of the *Amy32b *gene 5' area appears to be favourable for TBP binding, as it contains several inverted sequences [16], but it was shown that repeated sequences are often found in the TBP anchoring sites [3-6].

### TBP redistribution in the Bmy1 gene

In *Bmy 1 *gene, besides the overall decrease of TBP binding coupled to up regulation of transcription we have also observed drastic changes of TBP binding in Exon 3 and Intron 3, changes 1.5 Kb upstream the Exon 1 were also well-pronounced. Interestingly, Intron 3 of this gene harbours a microsatellite, MITE element, several other repeats and a predicted MAR (Figure 3). Both microsatellites and mobile elements are often involved in tight DNA-protein complexes [30,31] and hence these elements could also possess high affinity to TBPs. Indeed as position 10 of the microarray is very close to the microsatellite, sequences of the MITE element should hybridize with the oligonucleotide in the position 7. Enrichment of the mobile genomic elements in complexes with TBP was revealed also in our study on the characterization of the TBP-associated DNA sequences (unpublished results). On the other hand, the presence of a possible nuclear matrix attachment site (MAR) in Intron 3 of *Bmy 1 *gene and the absence of such sequences in other sites of the gene intensively binding TBPs indicate that TBP binding is not dependent on the presence of MAR.

### TBP binding and transcription

The overall intensity of the DNA association with TBP appears to be independent of transcription, as in stage 10 seeds we observed an overall decrease in the R vs F ratio along both genes, although one of them became expressed on this stage, but the other – remained dormant. Triggering or cessation of transcription is rather accompanied by redistribution of TBPs along the gene, but not overall accumulation or release of these proteins from the whole gene sequence. The same conclusion was made after a similar study on the chicken produced alpha-globin gene domain [7]. Comparison of TBP distribution in the domain in erythroid cells differing in the degree of alpha-globin gene transcription revealed changes in distribution of TBPs, however the TBPs were much more abundant along the domain in DNA from liver than in erythrocytes and erythroblasts. Thus, in animals overall enrichment in TBPs appears to be an organ-specific trait. Our results indicate that in plants it could be characteristic not only of the organ *per se*, but also of the development stage of an organ.

### Relation between TBP and nuclear matrix attachment sites

Application of the microsatellite analysis tool developed for the needs of marker-assisted selection during analysis of long-range DNA-protein interactions proved its utility especially for work with a partly sequenced genome like the barley genome. It should be mentioned that using the conventional PCR protocol we could observe only the most drastic changes in the sequence distribution of fractions; a quantitative real-time PCR technique would substantially increase the sensitivity of the approach. Nevertheless the assay enabled us to obtain interesting results. For example, the relation between TBP and nuclear matrix binding sites has been discussed for a long time and in some reports these are claimed to be identical structures [32-34], others consider TBP-DNA complexes to be specific structures, not related to nuclear matrix attachment sites [2,35]. Comparative study of the changes in distribution of complexes with TBPs and the nuclear matrix along Chromosomes 1H and 7H in the first leaf of the barley shoots indicates detachment of several sites in both chromosomes of the nuclear matrix during transition from watery to milky ripeness, at the same time the number of interaction with TBPs is increased (Figures 4 and 5). Thus, TBPs appear to form complexes different from the nuclear matrix association sites to DNA. Data from nucleoprotein celite chromatography are coherent with results obtained with salt-extracted nuclear matrices, but reveal the same process in more detail (Figures 5 and 6). The presence of a possible MAR only in one site of TBP accumulation also indicates the different nature of the two types of DNA-protein complexes.

### Individual peptides of TBP fraction

Detailed characterization of the TBP spectrum by means of 2D electrophoresis and mass-spectrometry are currently ongoing. However our preliminary results on development-dependent changes in TBP spectrum reported here are in good agreement with microsatellite analysis data. Indeed, we observe some increase in the TBP-binding sites during the transition of the first leaf from stage 07 to stage 10, the number of polypeptides in the TBP preparation increases, while in coleoptile we observed a decrease in the TBP binding sites and the peptide spectrum of TBPs is also simplified (Figures 7 and 8). Protein blot assays confirmed the same trend: in nuclear extracts of stage 07 coleoptiles several peptides bind TBP-associated DNA (Figure 8), in stage 10 coleoptiles the only low-molecular peptide, also prominent on electropherograms (Figure 7) retains its ability to form tight complexes with DNA.

## Conclusion

Our data indicate the existence of development-dependent redistribution of the TBP along *Amy32b *and *Bmy1 *and along studied stretches of Chromosomes 1H and 7H in barley. Sites of the TBP binding do not co-localize with the nuclear matrix attachment regions. The TBP spectrum and affinity to DNA change during plant development.

## Methods

### Plant material

Seeds of the barley cultivar *Balga *were obtained from the Latvian State Stende Cereal Breeding Institute and Latvian State Priekuli Crop Breeding Institute. Etiolated shoots were grown for 3 – 5 days at 30° in darkness. Coleoptiles, first leaves and roots were dissected from shoots of Zadoks 07 (coleoptile emerged stage, classification according [9]) and Zadoks 10 (first leaf through coleoptile) development stages. Dissected coleoptile and first leaf tissue from 50–100 shoots were combined in one sample for each tissue at both developmental stages. Ten embryos per sample from dry grains (Zadoks 0) and after 20 h of imbibition (Zadoks 1) were used in some experiments. Coleoptiles, first leaf, and embryo samples were subsequently used for bulk DNA extraction. Seeds of watery ripe (Zadoks 71) and medium milk development (Zadoks 75) stages were collected in the field of the Latvian State Priekuli Crop Breeding Institute. 70 – 100 mg of seed tissue was used for bulk DNA and RNA isolation.

### Genes

GenBank accession X05166 sequence information was used to develop primers and the microarray and to analyze data on the barley *Amy32b *gene [16]. GenBank accession AF061203 sequence information deposited for *Adorra *cultivar was used in this study for *Bmy1 *gene. The previously reported data [24] indicate the identity of the selected gene sequences in the *Adorra *and *Balga *cultivars used in this study.

### DNA and RNA isolation

Plant tissues were frozen in liquid nitrogen and ground in a mortar or in TissueLyser (Qiagen) to a fine powder. DNA from plant material was extracted according to the previously described protocol using chloroform-isoamyl alcohol extraction [36] with some modifications. Taking into account our previous observations on partial degradation of TBPs by proteases and the formation of artificial DNA-protein complexes due to the *RNaseA *treatment (reported also in [37]), we excluded the exogenous enzymes from DNA extraction protocols. RNA was precipitated with concentrated LiCl solution (up to 4 M) with subsequent 1 h incubation on ice [38] and removed as a pellet by centrifugation. DNA was precipitated with 1 volume of butoxyethanol. Nucleic acids from the NPC-chromatography fractions were concentrated by absorption on hydroxyapatite and elution with 1 ml of 0.24 M phosphate buffer. DNA was purified using a Wizard DNA Clean-Up System kit (Promega).

Total RNA was isolated according to the RNA extraction protocol of Rneasy Plant Mini Kit (Qiaqen). Samples of 2 μg – 3 μg each were stored at -20°C until use.

### Isolation of TBP-DNA complexes by means of fractionation on nitrocellulose

DNA was digested with *HindIII *and *Pst1 *restrictases or alternatively with *AluI *restrictase (1 U/1 μg of DNA) at 37°C overnight in appropriate buffer. For microarray experiments DNA was fragmented by sonication in a SONICS Vibra Cell™ device (Sonics & Materials, 53 Newtown, USA) with 60% output for a period of time ranging from 30 seconds to 4 minutes. Completeness of DNA digestion or extent of fragmentation was tested by gel electrophoresis. DNA samples with average fragment sizes of about 500 bp were diluted in 3 ml of filtration buffer (0.5 M KCl, 5 mM EDTA, 10 mM Tris- HCl, pH 7.4) to a final concentration of 25–70 μg/ml. The solution was pressed through a nitrocellulose filter (HAWP 025 00, HA 0.45 μ, 25 mm, Millipore or NC 45, 0.45 μm, 25 mm, Whatman (Schleicher & Schuell) pre-soaked with filtration buffer and supported in a Swinnex holder. The filter was washed up to five times with the same 3 ml volume of filtration buffer to avoid contamination between fractions. Filtration buffer wash off resulted in filtered DNA fraction (F). The filter retained a DNA fraction enriched in the tightly bound proteins; it was eluted with five washes of 3 ml each of 5 mM EDTA, 10 mM Tris HCl, pH 7.4 (low-ionic strength eluted fraction R1) and 3 ml of 50 mM NaOH (alkali-eluted fraction R2) in sequence [4]. The DNA content in the F, R1 and R2 fractions was measured spectrophotometrically.

### Chromatin fractionation and nuclear matrix isolation

A protocol of conventional extraction of nuclei with high salt solutions modified for monocots [39] was used for chromatin fractionation and nuclear matrix isolation. Briefly, nuclei isolated using 1% Triton X100 extraction were consecutively extracted with:10 mM Tris HCl, pH 7.5; 10 mM NaCl, 3 mM MgCl_2_; 20 mM NH_4_Cl to obtain the nucleoplasm fraction; with 10 mM Tris HCl, pH 7.5; 0.2 mM MgCl_2 _to obtain the fraction of soluble chromatin; and with 2 M NaCl, 10 mM Tris HCl, pH 7.5; 0.2 mM MgCl_2 _to obtain the fraction of insoluble chromatin. The residual fraction contained nuclear matrix.

### Chromatography of nucleoproteins on celite (NPC-chromatography)

The NPC-chromatography was performed as described [28,29]. Barley embryos or organs taken from 5 shoots were homogenized in a tightly-fit Dounce homogenizer in 10 mM Tris-HCl, pH7.6; 5 mM MgCl_2_, 1% Triton X100. The lysate was directly applied on a precooled (0°C) water-coated column of Celite R-630 (Fluka). The column was rinsed with 50 ml of 5 mM MgCl_2_, 10 mM Tris HCl, pH 7.4 (breakthrough fraction) at 0°C and 80 ml of NaCl in linearly increasing concentration (0 → 2 M) and the same temperature was pumped through the column. The fraction eluted in NaCl gradient (DNA 0) was considered to be unbound to the nuclear matrix. Then a gradient of LiCl – urea (0 → 4 M; 8 M) was applied in the same manner. Finally the column was gradually heated from 0°C to 100°C under a constant flow of 4 M LiCl, 8 M urea solution. Fractions eluted in LiCl-urea gradient and at a temperature below 70°C were combined (DNA I) and was considered to represent a fraction loosely bound to the nuclear matrix in contrast to the DNA II fraction eluted at high temperature (70° → 100°C) and considered to be tightly attached to the nuclear matrix.

### TBPs sample preparation and electrophoresis

Protein samples were obtained using the method of exhaustive DNase digestion [6]. The DNA was digested with *DnaseI *(Fermentas) (1 U/100 μg, room temperature, overnight) in 10 mM Tris-HCl, pH 7.6; 5 mM MgCl_2_. Completeness of digestion was monitored by gel electrophoresis. Proteins were concentrated by evaporation of excess water. The DNA digest was mixed with the sample buffer, heated to 100°C for 5 minutes and cooled. Electrophoresis was performed in 10% or 15% PAAG gels using the conventional protocol for Laemmli system. Gels were silver stained according to [40].

### PCR based marker chromosome profiling

Twenty two and twenty four primer pairs representing barley markers mapped along chromosomes 1H and 7H correspondingly were chosen for analysis (Figures 5, 6, 7). The primer sequences, genetic map position and microsatellite motif of HvALAAT, HVHVAI, Bmac, EBmac, Bmag and EBmag markers were previously published [24] and are publicly available . GBMS markers were developed and mapped in IPK [26]. The complete sequences of GBMS as well as WMCIE8, cMWG 773, AF022725A, cMWG 725, cMWG 728, ABG 461 markers are available from IPK on request. Markers of cMWG, ABC, and ABG series are primers of sequenced genomic RFLP clones [27], other markers are microsatellites.

To equalize the DNA content in different samples after fractionation on nitrocellulose, an aliquot of the F fraction was diluted 5 times, and aliquots of the R1 and R2 fractions were diluted twice. 5 μl of the diluted solution was taken for the amplification reaction. The final PCR reaction mix contained: about 100 ng of genomic DNA; 1.5 mM MgCl_2_, 40 μM of dCTP, dGTP, dTTP, dATP; 0.4 μM of each primer; and 1U of Taq polymerase in a 25 μl reaction mixture. The PCR conditions were: 3 min at 94°C, followed by 45 cycles at 94°C for 1 min, 60°C for 1 min, and 72°C for 2 min with chain elongation of 7 min [26]. PCR was performed on a Perkin Elmer PCR machine in 96-well format. Fragment length analysis was performed on an ALF II Express automated laser fluorescence sequencer (Pharmacia) using a short gel cassette. Denaturing polyacrylamide gels were prepared following the manufacturer's protocol (Pharmacia). The sample standard mix was heat denatured and snap cooled before loading. The gels were run in 0.5 × TBE buffer with 600 V, 50 mA and 50 W and gel temperature 50°C. Fragment sizes were calculated using the Fragment Analyser 1.02 computer program (Pharmacia) via comparison with the internal size standards (73, 122, 196, and 231 bp). Results were scored as presence/absence of amplification.

### DNA-binding protein blot assay

The assay was performed according to [41]. Nuclear proteins were electrophoretically separated in one dimensional 12% SDS-polyacrylamide gel. Proteins were electroblotted from the gel onto 0.45 μm nitrocellulose filters (Bio-Rad) in 19 mM glycine, 25 mM Tris, 0.1% SDS, and 20% methanol. Protein blots were blocked overnight at room temperature in blocking buffer (10 mM Tris-HCl, pH 7.5, 50 mM NaCl, 2 mM EDTA, 1% blocking reagent (BSA) and subsequently preincubated for 1 hour in binding buffer (10 mM Tris-HCI pH 7.4, 50 mM NaCl, 2 mM EDTA, 0.05% BSA). Later, the membranes were transferred into a fresh portion of the binding buffer supplemented with 10 ng/ml of radioactively labelled DNA probe (10,000–70,000 cpm/ml) and 100 ng/ml of competitor DNA (Eco R1 digested plasmid pUC19). TBP-bound DNA fragments obtained according to the exhaustive *DNaseI *digestion procedure were used as probes. Binding was carried out overnight at 37°C in a hybridization oven under gentle agitation. The membranes were washed three times for 15 min in 100 ml of binding buffer and autoradiographed.

### DNA probe labelling

Different DNA fractions were labelled with [α^32^P]dATP (Hartmann Analytic, Germany) using a HexaLabel™ DNA Labeling Kit (UAB Fermentas, Lithuania). Unincorporated nucleotides were removed by selective precipitation of DNA with ethanol in the presence of ammonium acetate.

### DNA array

*Amy32b *(GenBank accession X05166) and *Bmy1 *(GenBank accession AF061203) genomic sequence information was used to design the arrays. The DNA array for each gene was developed according to the published approach [7,42], and consisted of 50–60 oligonucleotides spaced along the whole gene. Sequences of the oligonucleotides are given in Table 1 and Table 2, their location in the structural genes is shown in Figures 2 and 3, for *Amy32b *and *Bmy1 *genes, respectively. The oligonucleotides had a similar T_m_. Prior to hybridization the oligonucleotides were analyzed *in silico *to avoid repetitive DNA sequences. The oligonucleotides were slot-blotted onto membrane Hybond Zeta Probe GT +0.4 M NaOH filters in 10 × SSC and fixed by baking at 80°C for 2 h. The hybridization was carried out at 65°C in modified Church buffer (0.5 M phosphate buffer, pH 7.2, 7% SDS, 10 mM EDTA) overnight. The blot was washed subsequently in 2 × SSC, 0.1% SDS twice for 30 min, then in 1 × SSC, 0.1% SDS for 30 min. The blots were exposed to Phosphorimager Fuji FLA-5100 for 3–12 h. The average of three independent experiments (two hybridizations per experiment) is presented. The signal of the total DNA hybridization was subtracted from the F and R fraction hybridization signal. Data are presented as R/F ratio of hybridization signals.

**Table 1:** *Amy32b *array oligonucleotide sequences.

Name	Sequence
1	5'-CCTCCCACGTTTATCTTCAATTTGTCAAAAAAATCATGTTCGGACCGTT-3'
2	5'-CAAAAGGTATATCCTGCGTAATATTTCTGTTACTGCACCACATTAAGAACAGTTTATATG-3'
3	5'-CAACGCTGGGTGATCCCAGCTTGGATAGTGCTATCTTTTCCCATGGAATTTGTGCCGGCC-3'
4	5'-TATCCATGCAGTGCCTCCAAGCAACACTCCACGGGGACGTAGCTCGTGTT-3'
5	5'-CAGTCTTGTGAATCATTCATCCACAGAACAAGAGTGCAGCGAACAGTGTAGATC G-3'
6	5'-CAACGAAGGTCCCTCTTCACACTAAAATCATTCGTGTCTCAACTGAACATC-3'
7	5'-AACGTGCAAATACGATCAAACAAGTATACAGTATACTGTACAAACTAAAAC-3'
8	5'-GACCACCTCAACGACCGCGTCCAGCGCGAGCTCAAGGAGTGGCTCCTCTGGCTCAAGAGC-3'
9	5'-CATGTGGCCATTCCCCTCCGACAAGGTCATGCAAGGCTACGCATACATCC-3'
10	5'-AATCATTCAGGAAACTAAAAATCTCTTGTCTTGTCGGTTTGCAGTTCTACGACCATTTCTTT-3'
11	5'-AAGCCTCCACTCATCCACCATTCAATCGAGCATGCATGAATTTTCCAAAATAATG-3'

**Table 2:** *Bmy1 *array oligonucleotide sequences.

Name	Sequence
1	5'-ACGTTTGAACATTAACGTGTGTTTTTGGTGAAAGTGAAAAAATAGTTGAC-3'
2	5'-ATAATTGGTGAGGCACATTCTCATTTGATTGGTTAGTTTAACTTCCTTGTCACATTATTT-3'
3	5'-AAAAAAAACAAATTTAGGATGATATTTTGGGGTAACTTTTGGTGTTCAATTTGTTTTTTT-3'
4	5'-ATGTGTGCGTCTTCACTTCGTATAGGGTGCCGTTTGGTTGAGAGTTGAGA-3'
5	5'-ACTATTTCAAGGATCTAGTGCACACATATACATTATTGTTGTACATATAACATTGATACT-3'
6	5'-CACCACTCTAGTTCTCTGATGCATATTTATATAGAAGTTCAAGATGACACCAAATACAAGC-3'
7	5'-TGGTGTTATCGTCGACATTGAAGTGGGACTTGGCCCAGCTGGAGAGATGAGGTACCCATC-3'
8	5'-AGAAATATATAGGATTCATCTGTGCAACTTAAATACTTAAAATGATTTTT-3'
9	5'-TTAAATTTTTAAATTGAGTGTCTTGGGTCTTGAATTTAAGACCTTTTGACTCGGATACCA-3'
10	5'-CTATGCATTTATACTTCAACAATAAGAATAGTGAGGTAGCAC-3'
11	5'-AACAACAAAAATACACAAAAC TATCCAGGCTAAGGGAACTCGCATTGCTTA-3'
12	5'-ATTTGTTGATTTGCAGGTGCCTATTATATACTAATAATTTAATTTTATTGTTTTCAGCCT-3'
13	5'-AGGCTGAAGGCCCCACCTGTGGCATGGGTGGGCAAGTTAAAGGCCCTACT-3'

### RT-PCR

RNA samples of 2 – 3 μg were pretreated with *DNaseI *using DNA-free kit (Ambion) and used for cDNA synthesis with Revert Aid™ H Minus First Strand cDNA Synthesis Kit (Fermentas) with unspecific primers according to the manufacturer's protocol. Aliquots containing 100 – 150 ng of the first strand cDNA were used as a template for PCR amplification performed in 30 μl of the reaction mixture with a final concentration of 1× PCR buffer (Fermentas), 3 mM of MgCl_2_, 40 μM of each dNTP, 1 μM of each forward and reverse sequence-specific primers and 0.612 U of Taq polymerase (Fermentas) per sample. Cycling parameters were as follows: denaturation/RT inactivation step at 94°C during 3 min was followed by 30 cycles of three steps; denaturation 45 sec at 94°C, annealing 45 sec at 60°C, and extension 1 min at 72°C, and final extension 5 min at 72°C. Primer design was performed by the Primer 3.0 program using coding regions of the genes. *Bmy1 *cDNA fragment of 178 bp in size was amplified with forward 5'-TGCCGTCCAGATGTATGC-3' and reverse 5'-GCAGATGAATTCTCCGATGC-3' primers to encompass part of exon II, the whole of exon III and part of exon IV. *Amy32b *cDNA fragment of 447 bp in size was amplified with forward 5'-TAAGCCCAACTACGATCAGGA-3' and reverse 5'-ACGTAGGCGTCTCCTTCGTG-3' primers to encompass partly exon III and exon IV. Sequences of primers for the alpha tubulin (*Tub*) reference gene, a ubiquitous and stably expressed gene in barley, have been published [43]. Amplification resulted in the 248 bp product of expected size.

### Data management and analysis

Numbering of any loci in the genes is given from the first ATG of the corresponding genomic sequence. Identification of transcription factor binding sites and clusters, analysis of similarities between the two genes and prediction of nuclear matrix attachment regions (MAR) was performed by Genomatix software (DiAlign TF, Release 3.1, and MatInspector, Release 7.4, Smartest Release 2.2) tools at ).

## Abbreviations

TBP: tightly bound protein; TBPs: tightly bound proteins; NM: nuclear matrix; SC: soluble chromatin; IS: insoluble chromatin; MS: microsatellite; NPC-chromatography: nucleoprotein-celite chromatography.

## Authors' contributions

TS initiated, coordinated, and led the project, performed PCR-based marker and bionformatical analysis and edited the manuscript. NS designed the study, analyzed the results of all the experimental work described and drafted the manuscript. MR worked out the strategy of marker analysis. YV conceived and designed the microarray experiments performed by KB. OS performed RT PCR experiments. LB, DL, KB performed experiments with proteins designed by BJ. All the authors read and approved the final manuscript.
